# Pathogenic and non-pathogenic *Escherichia coli* colonization and host inflammatory response in a defined microbiota mouse model

**DOI:** 10.1242/dmm.035063

**Published:** 2018-11-16

**Authors:** Zachary R. Stromberg, Angelica Van Goor, Graham A. J. Redweik, Meghan J. Wymore Brand, Michael J. Wannemuehler, Melha Mellata

**Affiliations:** 1 Department of Food Science and Human Nutrition, Iowa State University, Ames, IA 50011, USA; 2 Department of Veterinary Microbiology and Preventive Medicine, Iowa State University, Ames, IA 50011, USA

**Keywords:** *E*. *coli*, Enterohemorrhagic *E*. *coli*, Inflammation, Mouse model

## Abstract

Most *Escherichia coli* strains in the human intestine are harmless. However, enterohemorrhagic *E*. *coli* (EHEC) is a foodborne pathogen that causes intestinal disease in humans. Conventionally reared (CONV) mice are inconsistent models for human infections with EHEC because they are often resistant to *E*. *coli* colonization, in part due to their gastrointestinal (GI) microbiota. Although antibiotic manipulation of the mouse microbiota has been a common means to overcome colonization resistance, these models have limitations. Currently, there are no licensed treatments for clinical EHEC infections and, thus, new tools to study EHEC colonization need to be developed. Here, we used a defined microbiota mouse model, consisting of the altered Schaedler flora (ASF), to characterize intestinal colonization and compare host responses following colonization with EHEC strain 278F2 or non-pathogenic *E*. *coli* strain MG1655. Significantly higher (*P*<0.05) levels of both strains were found in feces and cecal and colonic contents of C3H/HeN ASF compared to C3H/HeN CONV mice. GI inflammation was significantly elevated (*P*<0.05) in the cecum of EHEC 278F2-colonized compared to *E. coli* MG1655-colonized C3H/HeN ASF mice. In addition, EHEC 278F2 differentially modulated inflammatory-associated genes in colonic tissue of C3H/HeN ASF mice compared to *E. coli* MG1655-colonized mice. This approach allowed for prolonged colonization of the murine GI tract by pathogenic and non-pathogenic *E*. *coli* strains, and for evaluation of host inflammatory processes. Overall, this system can be used as a powerful tool for future studies to assess therapeutics, microbe-microbe interactions, and strategies for preventing EHEC infections.

## Abstract (precis)

**Summary:** Mice harboring a defined microbiota of the altered Schaedler flora were identified as a reliable tool to assess prolonged *Escherichia coli* intestinal colonization and activation of the host inflammatory response.

## INTRODUCTION

The mammalian gastrointestinal (GI) tract harbors a vast density and diversity of microorganisms that can provide protection against pathogen colonization. *Escherichia coli* is one of the first members to colonize infants and establishes as a life-long resident of the normal intestinal microbiota in humans (Eggesbø et al., 2011). Non-pathogenic *E*. *coli* strains provide the host benefits such as vitamin K and B_12_ (Blount, 2015); however, certain *E*. *coli* strains can cause disease. Enterohemorrhagic *E. coli* (EHEC) is a pathotype of diarrheagenic *E*. *coli*, which causes mild to severe bloody diarrhea in humans that can progress to hemolytic uremic syndrome (Croxen et al., 2013). EHEC strains carry genes for Shiga toxin and the locus of enterocyte effacement (LEE), including the type III secretion system and major adherence factor intimin (Feng et al., 1998; Stevens and Frankel, 2014). In the US, O157:H7 EHEC is the most common serotype associated with EHEC infections in humans (Gould et al., 2013). Ruminants, including cattle, are the primary reservoir for O157 EHEC (Gyles, 2007), and the pathogen is commonly transmitted to humans by direct contact with ruminants or through ingestion of fecally contaminated food or water (Cole et al., 2014). After ingestion, EHEC colonizes colonic epithelial cells and carries both inflammatory stimulators (e.g. endotoxin, flagellin, Shiga toxin) and suppressors (e.g. type III secreted effectors) (Pearson and Hartland, 2014). O157 EHEC colonization and presentation of disease symptoms typically occurs within 1-2 weeks after ingestion (Tarr et al., 2005). Children under 5 years old are more likely to develop severe disease following infection with EHEC (Croxen et al., 2013). A few studies have determined that the shedding duration of O157 EHEC in children can last over 100 days, with most reporting a mean duration of 1 month (Miliwebsky et al., 2007; O'Donnell et al., 2002; Shah et al., 1996). Adults may also develop severe disease but, in some adults, such as cattle farm and processing workers, asymptomatic colonization with EHEC has been reported (Hong et al., 2009; Silvestro et al., 2004).

Animal models to study *E*. *coli* have limitations in establishing intestinal colonization. Large animal models such as baboons (Taylor et al., 1999), greyhounds (Raife et al., 2004), monkeys (Kang et al., 2001) and pigs (Francis et al., 1986, 1989; Tzipori et al., 1986, 1992) have poor scalability and require extensive veterinary expertise, labor and funding. Mice serve as an attractive model organism due to their size, short generation time and characterized genetics. However, conventionally reared (CONV) adult mice are often naturally resistant to *E. coli* colonization (Roxas et al., 2010). To overcome resistance, streptomycin has been used to suppress facultative anaerobic bacteria, allowing for streptomycin-resistant *E*. *coli* to colonize (Allen et al., 2006; Leatham et al., 2009; Lindgren et al., 1993). Streptomycin-treated mice have a variable microbiota and require generation of streptomycin-resistant *E. coli* mutants, which can affect expression of bacterial virulence factors (Chen et al., 2013). Other studies have used germ-free mouse models to study *E*. *coli* colonization (Goswami et al., 2015; Taguchi et al., 2002), but these animals lack the influence of a microbiota and have underdeveloped mucosal immune systems and altered intestinal morphology (Hooper et al., 2012). A few studies have used CONV mouse models, but administration of a high dose [≥10^9^ colony-forming units (CFU)] is required and variable colonization was observed (Mohawk et al., 2010; Nagano et al., 2003). Thus, developing reliable animal models to study *E*. *coli* intestinal colonization remains of utmost importance.

The altered Schaedler flora (ASF) is a set of eight bacterial species that can be stably maintained in gnotobiotic mice over many generations (Wymore Brand et al., 2015). ASF mice develop normal GI function and immune systems (Geuking et al., 2011; Sarma-Rupavtarm et al., 2004). Previously, it was demonstrated that, without the use of antibiotics, adherent and invasive *E. coli* colonized C3H/HeN ASF mice (Henderson et al., 2015) and *Salmonella enterica* colonized C57BL/6 ASF mice (Stecher et al., 2010). In addition, the adherent invasive *E*. *coli* strain LF82 induced severe colitis in ASF mice exposed to dextran sodium sulfate (Henderson et al., 2015). In the current study, ASF-colonized C3H/HeN mice were used to assess intestinal colonization and the host inflammatory response to non-pathogenic *E*. *coli* and EHEC inoculation. The objectives of this study were to: (1) determine the extent of *E*. *coli* colonization in the small and large intestine; and (2) compare the host intestinal response to EHEC versus non-pathogenic *E*. *coli* colonization.

## RESULTS

### Characterization of EHEC strain 278F2

Major virulence genes intimin (*eae*) and Shiga toxin (*stx*) were tested for by using PCR assays. The clinical O157:H7 strain 278F2 was confirmed to be positive for both *eae* and Shiga toxin type 2 (*stx*_2_). EHEC strain 278F2 was further subtyped as Shiga toxin subtype 2a (*stx*_2a_)-positive by amplicon sequencing and VirulenceFinder analysis, and confirmed using subtype-specific primers (Joensen et al., 2014; Scheutz et al., 2012).

### Concentration of *E. coli* in ASF and CONV mice

**Fig. 1.**
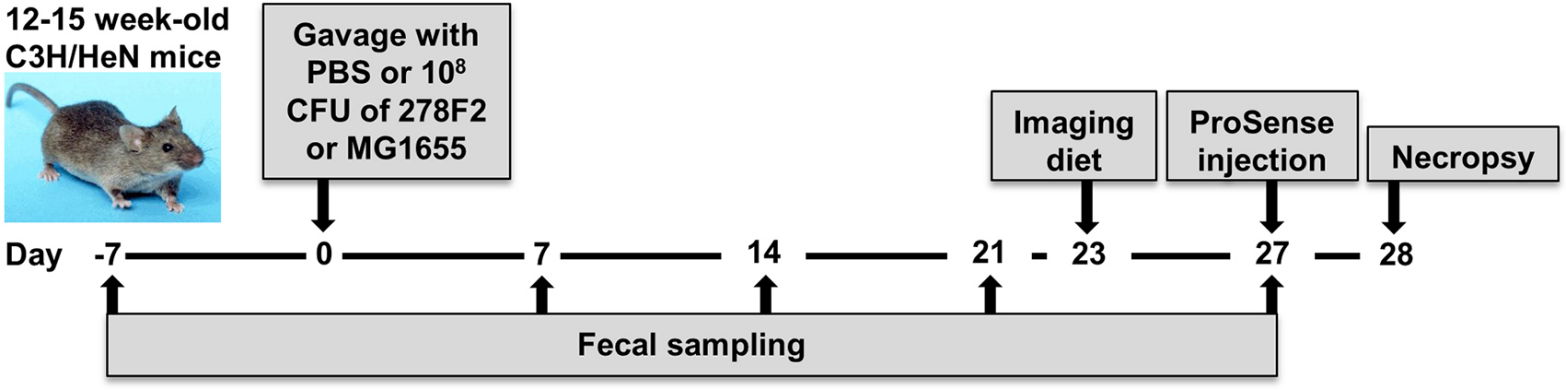
**Timeline of experimental procedures.** Mice were inoculated with PBS (control) or 10^8^ CFU of enterohemorrhagic *E*. *coli* (EHEC) 278F2 or non-pathogenic *E. coli* MG1655. Weekly fecal samples were collected as indicted. The diet was changed to Teklad 2920X for fluorescent imaging and mice harboring the altered Schaedler flora (ASF; *n*=3 per group) were injected on day 27 post-inoculation with ProSense 680. At necropsy, the gastrointestinal (GI) tract was excised and imaged for mucosal inflammation (ASF mice only), intestinal contents were collected for enumeration of *E*. *coli* [ASF and conventionally reared (CONV) mice], and intestinal tissues were harvested (ASF mice only) for histopathology and host transcriptome profiling.

**Fig. 2.**
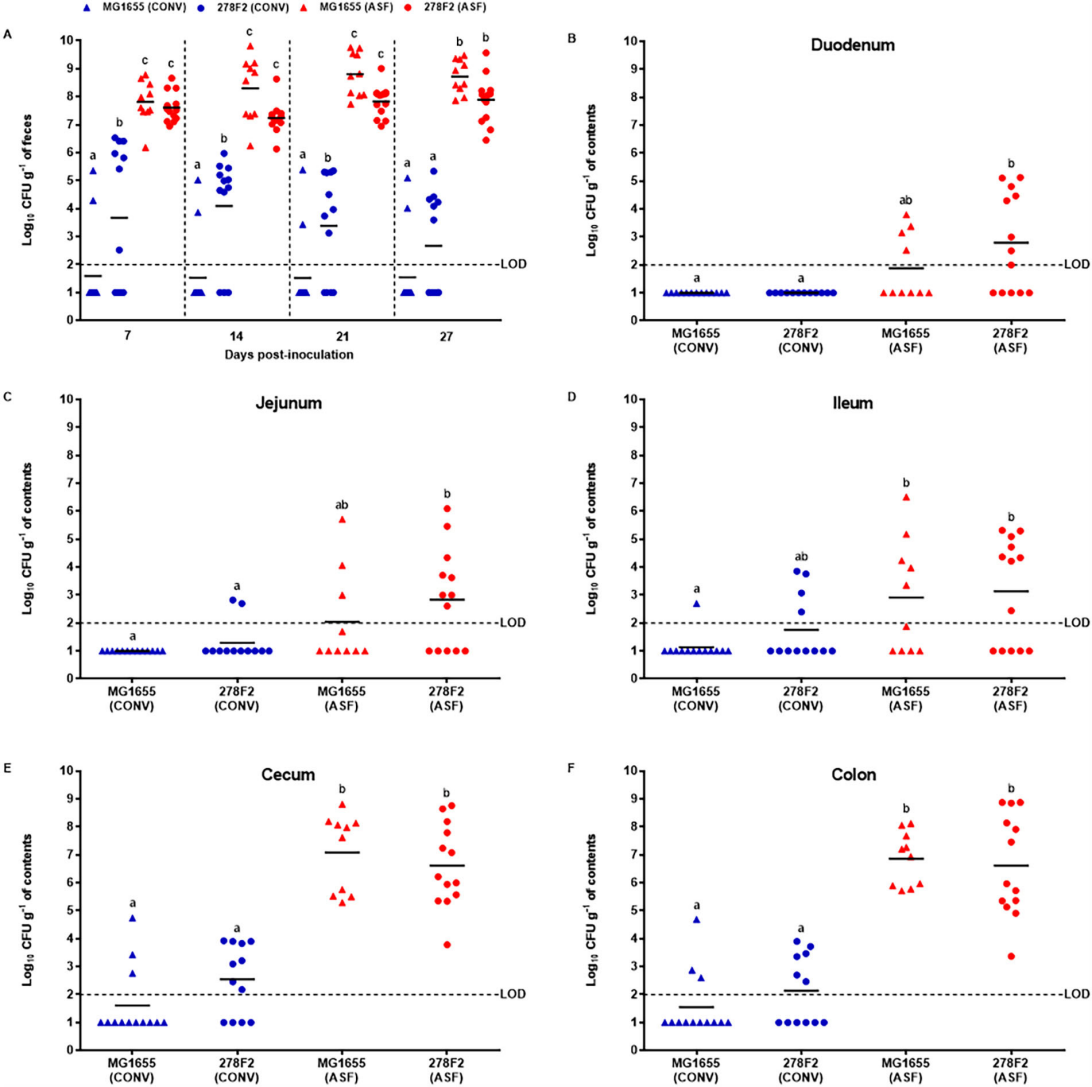
**Ability of *E. coli* to colonize mice harboring the altered Schaedler flora (ASF) or a conventionally reared (CONV) microbiota.** Mice were inoculated with 10^8^ CFU of pathogenic enterohemorrhagic *E*. *coli* (EHEC) strain 278F2 or non-pathogenic strain MG1655. The ability of strains to colonize the gastrointestinal tract was assessed by collecting (A) fecal samples on days 7, 14, 21 and 27 post-inoculation and (B-F) intestinal contents on day 28 post-inoculation. The sample size for each group was as follows: 278F2- or MG1655-inoculated ASF mice (*n*=13 or 10, respectively) and 278F2- or MG1655-inoculated CONV mice (*n*=12 or 13, respectively). Data are representative of at least two individual experiments. Each symbol represents an individual animal and bars indicate the mean. Blue and red symbols represent CONV and ASF mice, respectively. Means with the same letter are not significantly different (*P*>0.05) as determined by an ANOVA followed by Tukey's test for multiple means comparison. The horizontal dashed line is the limit of detection (LOD). Samples that tested negative for *E*. *coli* were assigned a value halfway between zero and the LOD.

To determine the ability of *E. coli* strains to colonize and persist in the GI tract of ASF and CONV mice, fecal samples were collected at 7, 14, 21 and 27 days post-inoculation, and intestinal contents were collected on day 28 post-inoculation (Fig. 1). All mice survived the length of the study and no *E*. *coli* was detected in uninfected ASF and CONV mice. Overall, *E*. *coli* strains were detected in the feces and consistently recovered from cecal and colonic contents of ASF mice at significantly higher (*P*<0.05) concentrations compared to CONV mice (Fig. 2). Concentrations of EHEC strain 278F2 and non-pathogenic *E*. *coli* MG1655 isolated from fecal samples of ASF mice ranged from 10^6^ to 10^9^ CFU g^−1^ and were significantly higher (*P*<0.05) on all days tested compared to CONV mice, which had levels ranging from not detected to 10^6^ CFU g^−1^. When comparing colonization levels within CONV mice, 278F2 was detected at significantly higher (*P*<0.05) levels than MG1655 in feces on days 7, 14 and 21, but not on day 27. In CONV mice, 278F2 was detected in fecal samples from six to nine of 12 mice depending on the sampling day, while MG1655 was detected in only two of 13 CONV mice. In small-intestinal contents of ASF mice, 278F2 was significantly higher (*P*<0.05) compared to 278F2 and MG1655 in CONV mice, with the exception of 278F2 in the ileum of CONV mice. In the cecum and colon of ASF mice, 278F2 and MG1655 were in quantifiable concentrations ranging from 10^4^ to 10^8^ CFU g^−1^ and were significantly higher (*P*<0.05) than in CONV mice, which had *E*. *coli* at levels ranging from not detected up to 10^4^ CFU g^−1^.

### Body weight changes of ASF and CONV mice

Body weight was measured weekly and percentage change from that prior to oral gavage was calculated (Table S1). Groups within day 7, 14, 21 and 27 were compared for mean body weight and mean percent change in body weight. *Escherichia coli* colonization did not cause any significant differences within groups of ASF or CONV mice, but CONV mice did gain significantly more weight (*P*<0.05) compared to ASF mice. Colonization of ASF mice with 278F2 or MG1655 induced weight loss at day 7. However, the percent change in body weight of 278F2-inoculated versus uninfected ASF mice was not significantly different. *Escherichia coli* 278F2-inoculated ASF mice gained 3.4% and 5.7%, while uninfected ASF mice had an increase of only 1.9% and gained 3.0% on days 14 and 21, respectively.

### Impact of *E*. *coli* strains on tissue damage in ASF mice assessed by histological grading

Formalin-fixed intestinal tissue sections from uninfected and MG1655- or 278F2-colonized ASF mice were processed and scored for mucosal height, epithelial injury, inflammation and lesion presence. Summarized histological scores and measurements from five mice per group are shown in Table 1. No significant differences were found between groups of mice inoculated with different *E*. *coli* strains for histopathological scoring of ileal and colonic tissue. Colonic lesions were observed in one of five MG1655-colonized mice, displaying increased inflammatory cells, and two of five 278F2-colonized mice, with one exhibiting increased inflammatory cells and the other showing elongation of the glands (Fig. S1).

**Table 1.**
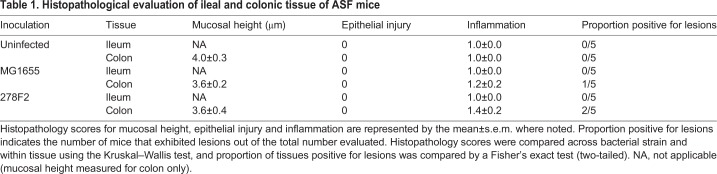
**Histopathological evaluation of ileal and colonic tissue of ASF mice**

### Evaluation of inflammation in the intestine of ASF mice

**Fig. 3.**
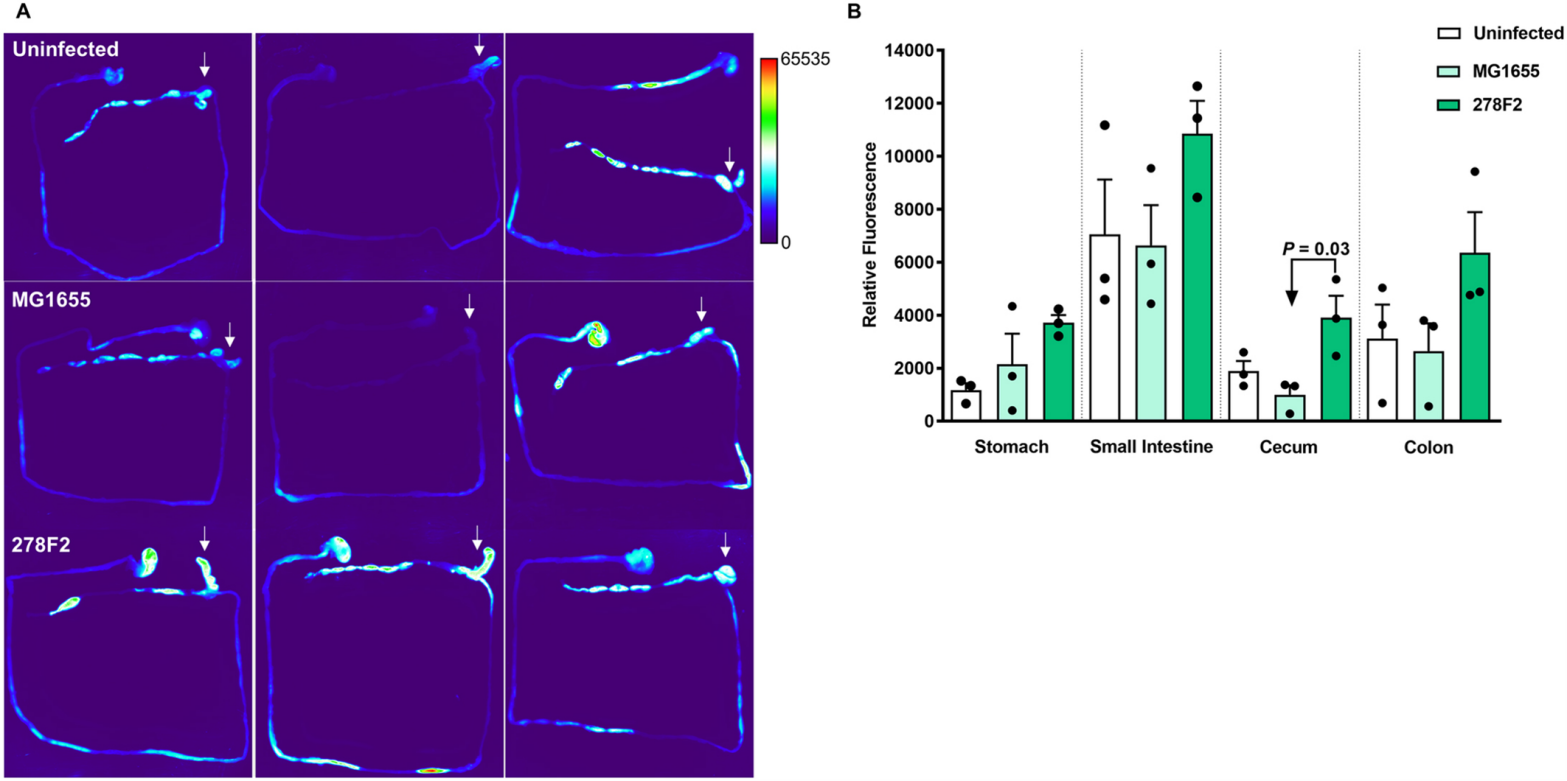
**Evaluation of mucosal inflammation in mice with a microbiota consisting of the altered Schaedler flora (ASF).** Mice received a tail vein injection with 2 nmol per mouse of ProSense 680 and were imaged 18 h later using the In Vivo Multispectral Imaging System FX Pro. (A) The entire GI tract was excised from uninfected (top row), non-pathogenic *E. coli* MG1655-inoculated (middle row), and enterohemorrhagic *E*. *coli* (EHEC) 278F2-inoculated (bottom row) mice (*n*=3 per group). Arrows indicate the cecum. (B) Relative fluorescence was measured in ImageJ. An ANOVA followed by Tukey's method for multiple means comparison was used to compare total corrected cellular fluorescence of intestinal sections. Each symbol represents an individual animal and bars represent mean±s.e.m.

To assess the effect of *E. coli* strains on mucosal inflammation, ASF mice were injected with ProSense 680, which is a fluorescent probe used to detect inflammation-associated protease activity. Heat-map images of the GI tract from uninfected or *E*. *coli-*colonized mice were generated and analyzed for total corrected cellular fluorescence (TCCF) (Fig. 3). Mucosal inflammation in ASF mice colonized with 278F2 was significantly higher (*P*=0.03) than in MG1655-colonized mice in the cecum (Fig. 3B). No significant differences were found between groups for the stomach, small intestine or colon. However, ASF mice colonized with 278F2 had numerically higher mucosal inflammation levels compared to both uninfected and MG1655-colonized mice in all regions tested.

### Comparison of cytokine levels released from colonic biopsies

The levels of interleukin (IL)-1β, IL-12, interferon gamma (IFNγ) and tumor necrosis factor alpha (TNFα) released from colonic explants was assessed to determine whether *E*. *coli* strains elicited a proinflammatory environment. For colon biopsies of 278F2 inoculated ASF and CONV mice, cytokine levels were numerically higher than uninfected mice, but were not significantly different (*P*>0.05) (Fig. S2). IL-12 was significantly higher (*P*<0.05) in biopsies of MG1655-inoculated CONV mice compared to uninfected mice.

### Comparison of inflammatory-associated gene expression and protein-protein interactions in the colon of ASF mice

To further characterize the host response to colonization with *E*. *coli* strains, a targeted gene expression panel was used to identify differentially expressed genes (DEGs) between ASF mice uninfected or infected with different strains of *E. coli*. The colon was selected because it is the major site of *E*. *coli* colonization in humans; the highest levels of 278F2 and MG1655 were recovered from the colon of mice in the present study (Fig. 2), and some mice exhibited colonic lesions (Fig. S1; Table 1). High-quality RNA was used for RNA-seq, with an average RNA integrity number of 9.1 (Table S2). The results of gene expression in the colon for the comparisons of uninfected versus MG1655-colonized, uninfected versus 278F2-colonized and MG1655-colonized versus 278F2-colonized ASF mice are found in Table 2. Twenty-seven DEGs were detected in the comparison of MG1655-colonized versus uninfected mice, with 23 upregulated and four downregulated genes. Eighteen DEG were detected in the comparison of 278F2-colonized versus uninfected mice, with 13 upregulated and five downregulated. Five genes were differentially expressed in both 278F2- and MG1655-colonized mice compared to the uninfected group. Of these five genes, three (*Cxcl9*, *Il17f* and *Inhba*) were upregulated in both 278F2- and MG1655-colonized mice, while the two remaining DEGs (*Cebpa* and *Cxcl10*) were inversely regulated relative to each other. For the comparison of 278F2- versus MG1655-colonized ASF mice, 17 DEGs were detected, with ten upregulated and seven downregulated. The largest fold-change increase was in Fc receptor, IgE, high affinity I, alpha polypeptide (*Fcer1a*) and the largest decrease was identified in urotensin 2 (*Uts2*).

**Table 2.**
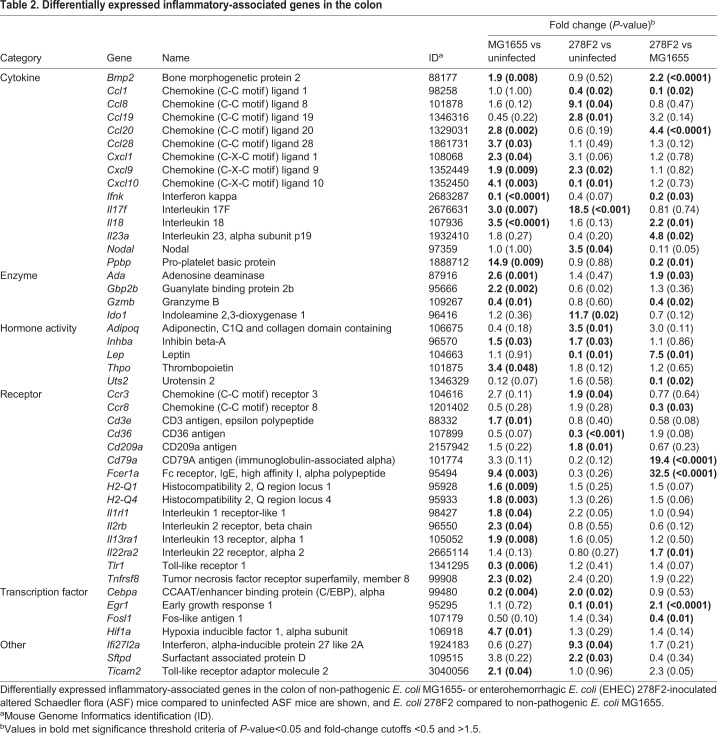
**Differentially expressed inflammatory-associated genes in the colon**

**Fig. 4.**
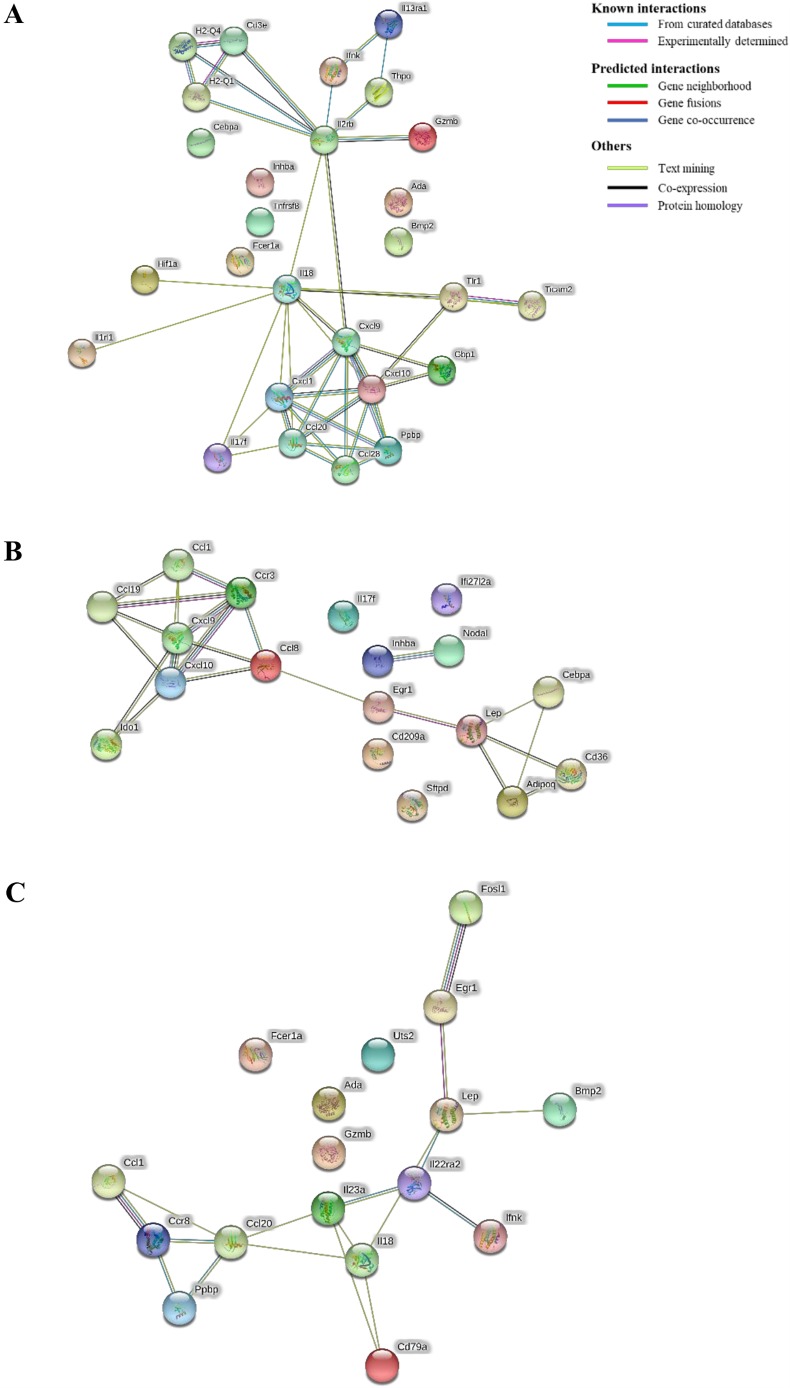
**Protein-protein interaction network among differentially expressed genes (DEGs) in colon tissue of altered Schaedler flora (ASF) mice.** DEGs from the comparisons of (A) uninfected vs MG1655-colonized, (B) uninfected vs 278F2-colonized and (C) MG1655-colonized vs 278F2-colonized ASF mice were used as input for protein-protein interactions. The edges connecting proteins represent the predicted functional associations and are colored based on the type of interaction.

In the protein-protein interaction network comparisons of uninfected versus MG1655-colonized (Fig. 4A) and uninfected versus 278F2-colonized ASF mice (Fig. 4B), the strongest clustering was between the chemokine ligands, with six chemokines in each cluster per comparison. The former revealed Il2rb as a hub, with connections to eight other proteins (Fig. 4A), and the latter revealed Lep as a hub, with connections to four other proteins (Fig. 4B). In the comparison of 278F2- versus MG1655-colonized ASF mice (Fig. 4C), the chemokine cluster was smaller than the other comparisons and only contained three proteins (Ccl1, Ccl20 and Ccr8). The Ccl20 protein had connections to five other proteins, making it the biggest hub for the comparison of MG1655- versus 278F2-colonized ASF mice.

## DISCUSSION

*Escherichia coli* is one of the best-understood microorganisms, but we know little about its impact on the intestine and which microbe-microbe interactions influence colonization *in vivo*. To study *E*. *coli* in the GI tract, ASF mice harboring a stable eight-member microbiota were characterized for: colonization compared to CONV mice, mucosal inflammation and expression of inflammatory-associated genes after colonization with a pathogenic or non-pathogenic *E*. *coli* strain. Stable colonization of ASF mice was achieved using a single gavage of 10^8^ CFU (Fig. 2). Concentrations of both strains increased along the GI tract, as assessed from the duodenum to the colon, in ASF mice (Fig. 2), similar to that observed in humans (Scheithauer et al., 2016). It is difficult to directly compare our study with other mouse models, as varying bacterial and mouse strains, mouse age, microbiota, and inoculum concentration likely contribute to reported differences in *E. coli* colonization. Earlier studies have used MG1655 as a non-pathogenic *E*. *coli* to study colonization of streptomycin-treated mouse models (Conway and Cohen, 2015; Gauger et al., 2007; Leatham et al., 2009). In those studies, MG1655 and the EHEC strain EDL933 were found to occupy distinct nutritional niches in the mouse intestine (Conway and Cohen, 2015). Although MG1655 lacks many of the virulence and colonization factors found in EHEC, it may be able to persist in ASF mice due to an open nutritional niche. Future studies could explore the open nutritional niches found in ASF mice that are not found in CONV mice. Although the use of CONV mice is advantageous for assessing interaction and competition with a complex microbiota, we found that some CONV mice were not colonized by 278F2 (Fig. 2A) and only two CONV mice had consistent recovery of MG1655 in feces (Fig. 2A) using our method of detection. In a different study using CONV mice, inoculation with approximately 10^9^ CFU of EHEC resulted in rapidly reduced levels (3-4 log_10_) within 24 h (Mohawk et al., 2010). In another study using CONV mice, inoculation with a higher level (10^10^ CFU) resulted in similar clearance of EHEC, and a shedding duration of only 2 weeks was observed for most mice (Conlan and Perry, 1998). While it is unclear how long ASF mice could be colonized, we showed that these mice shed *E*. *coli* for at least 1 month (Fig. 2), which mimics the mean duration of O157 EHEC shedding in children under 5 years old (Miliwebsky et al., 2007; O'Donnell et al., 2002; Shah et al., 1996).

It is thought that facultative anaerobes in CONV mice inhibit growth of invading *E*. *coli* strains. Therefore, streptomycin treatment is commonly used to suppress facultative anaerobes to allow *E*. *coli* to overcome colonization resistance in mice (Chen et al., 2013; Leatham et al., 2009; Mohawk and O'Brien, 2011). The exact mechanism of reduction of colonization resistance after streptomycin administration is unclear, but elimination of microbe-microbe interactions and a streptomycin-induced inflammatory response to support growth of *E*. *coli* through nitrate respiration has been suggested (Spees et al., 2013). A limitation of using spontaneous streptomycin-resistant *E*. *coli* strains is that they may have slight to significant decreases in colonization factor expression that could influence host-pathogen interactions (Chen et al., 2013). In addition, streptomycin treatment alone leads to a modest increase in histopathological changes, making the intestine more irritable (Spees et al., 2013). Germ-free mouse models have also been used (Goswami et al., 2015; Taguchi et al., 2002), but these models lack the ability to assess microbe-microbe interactions and have underdeveloped mucosal immune systems (Hooper et al., 2012). Thus, ASF mice serve as an alternative to germ-free and streptomycin-treated models, and can be used to assess inflammatory changes without the use of a confounding antibiotic treatment.

Inflammation in the GI tract plays a key role in defending against pathogens. Bacterial components such as endotoxin, flagellin and Shiga toxin can stimulate the inflammatory response during EHEC infection (Pearson and Hartland, 2014). The concentrations of common proinflammatory markers, including IL-1β, IL-12, IFNγ and TNFα, were numerically greater in colon supernatants of EHEC-inoculated mice compared to uninfected mice (Fig. S2), but no significant differences were found. These results indicate that other factors may account for intestinal inflammation observed in this system. However, IL-12 was significantly higher (*P*<0.05) in supernatants of MG1655-inoculated CONV mice compared to uninfected mice. IL-12 is produced by dendritic cells and macrophages in response to microbial products binding to Toll-like receptors (Trinchieri, 2003). In addition, mucosal inflammation was assessed using a technique that relied on injection of an imaging agent that is activated in the presence of inflammation-associated proteases such as cathepsin B, K, L and S. Here, elevated levels of mucosal inflammation were found in the cecum of 278F2- compared with MG1655-colonized ASF mice (Fig. 3), which is consistent with gene expression data showing increased chemokine expression (e.g. *Ccl20*) that could recruit macrophages to the mucosa. Although both the cecum and colon had high levels of EHEC, previous studies have found that EHEC closely associates with the cecal epithelium and not the colon in mice (Eaton et al., 2008; Roxas et al., 2010). This possibility may account for why a significant difference was observed only in the cecum. Previously, cathepsin B release was shown in EHEC-infected human monocytes but could not be demonstrated using mouse monocytes (Cheng et al., 2015). This suggests that other cathepsins may be released or other cell types may release cathepsin B in the mouse during EHEC infection. The presence of cathepsins, primarily macrophage enzymes, at 28 days post-inoculation indicates elicitation of chronic inflammation. Infectious gastroenteritis caused by zoonotic pathogens has been proposed as a risk factor in the ‘multi-hit’ model of inflammatory bowel disease (Gradel et al., 2009; Small et al., 2016). Thus, both the acute infection phase and the chronic impact of GI inflammation pose risks to human health. To counteract inflammation, EHEC releases effector proteins (Pearson and Hartland, 2014). EHEC strains inhibit NF-κB activity in a type-III-secretion-system-dependent manner (Hauf and Chakraborty, 2003). Previously, multiple non-LEE effector proteins were found to interfere with innate immune system signaling pathways (Gao et al., 2009; Nadler et al., 2010). Thus, while host cells may recognize microbial factors and activate inflammatory pathways to eliminate the pathogen, EHEC uses strategies to interfere with these signals to survive in the inflamed intestine.

Various levels of disease, from no apparent change to death, have been reported in *E*. *coli-*colonization mouse models. In the current study, only modest histopathological changes such as increased inflammatory cells and elongation of the glands were identified (Fig. S1, Table 1), and no mice died. Colonic lesions have been found in other mouse models of EHEC infection (Békássy et al., 2011; Brando et al., 2008) and were most thoroughly described in a germ-free model (Eaton et al., 2017). Previous studies using EHEC mouse models have noted weight loss (Mohawk et al., 2010) but, in the current study, only a slight initial decrease was observed for MG1655- and 278F2-colonized ASF mice (Table S1). In addition, both ASF and CONV mice inoculated with 278F2 gained weight on all but 7 days post-inoculation. Changes in inflammatory-associated genes that are also linked to metabolic processes, such as adiponectin, leptin and thrombopoietin, indicate that metabolic processes were modulated after chronic colonization with 278F2, which may account for the gain in body weight. CONV mice had significantly greater changes in body weights compared to ASF mice on 21 and 27 days post-inoculation, except for 278F2-colonized ASF mice on day 21. Previously, Bäckhed et al. (2004) found that CONV mice had significantly higher body fat content compared to germ-free mice, suggesting that microbiota influences weight gain. In addition to differences in weight gain, oral inoculation with O157 EHEC has been lethal in some mouse models. In one study, 40% of C3H/HeN mice died after intragastric inoculation with 10^8^ CFU of EHEC O157:H7 (Karpman et al., 1997). In another study, 100% of immature BALB/c mice died within 24 h using an inoculum of 10^10^ CFU kg^−1^ (Brando et al., 2008). Death was likely due to translocation of Shiga toxin into the systemic circulation after intestinal tissue damage in these lethal models. Therefore, mature ASF mice can be used as a stable non-lethal model in future studies to assess the efficacy of vaccines for reducing fecal shedding of EHEC and further explore microbe-microbe interactions.

ASF mice have not been exposed to *E*. *coli* or any other Proteobacteria, allowing for the first interactions between *E*. *coli* and host to be studied. A targeted approach (*N*=500 genes) was used to characterize the effect of prolonged *E*. *coli* colonization on host gene expression in the colon. To our knowledge, this is the first study to characterize a large set of inflammatory-associated genes in response to EHEC colonization in a mouse model. Most studies have focused on bacterial gene expression, while those targeting host expression have been limited to human cell lines infected for less than 24 h (Berin et al., 2002; Ceponis et al., 2003; Jandu et al., 2006; 2007; Karve et al., 2017; Kim et al., 2009). In the current study, colons were removed 28 days after inoculation to mimic the average duration of O157 EHEC shedding in children. EHEC colonization produced a distinct gene expression pattern (Table 2), which was expected considering the large differences between non-pathogenic *E*. *coli* K-12 and O157 EHEC strains (Hayashi et al., 2001). In the comparison between 278F2- and MG1655*-*colonized ASF mice, 41% of the DEGs were downregulated, including cytokines *Ccl1*, *Ifnk* and *Ppbp* and cytokine receptor *Ccr8*, giving insight into mechanisms used by 278F2 to modulate host immunity (Table 2). Another downregulated gene, *Gzmb*, is involved in apoptosis of infected cells, which may be an important aspect in the ability of EHEC to evade host immunity. Increased expression of cytokines (*Bmp2*, *Ccl20*, *Il18*, *Il23a*) in 278F2- compared to MG1655-colonized mice was consistent with increased inflammation observed histologically and by intestinal imaging after ProSense injection. Future studies may use RNA-seq to characterize the host transcriptome, which includes all genes rather than those already known to be involved in immunity. Also, subsequent studies could use RNA-seq to compare ASF to CONV mice to determine how microbiota impacts host response after *E*. *coli* inoculation. Furthermore, understanding global gene expression patterns in the intestine that are affected by *E*. *coli* may be increasingly important, as early infant colonization with commensal *E*. *coli* has recently declined in Western countries (Adlerberth et al., 2006; Eggesbø et al., 2011).

Protein-protein interactions are at the core of cellular responses, including response to infection. In the current study, chemokine interactions were the largest networks in all comparison groups, likely due to their myriad of functions during infection, e.g. chemotaxis, and leukocyte activation and extravasation (Mahalingam and Karupiah, 1999). In 278F2- compared to MG1655-colonized mice, the clustering showed interesting predictions for mechanisms of downregulation during EHEC infection. The downregulated DEGs *Ccl1*, *Ccr8* and *Ppbp* were all associated in the protein network (Fig. 4A). Previous research has shown that Ccl1 and Ccr8 are co-expressed in macrophages (Szebeni et al., 2017). Furthermore, Ppbp is predicted to activate expression of Ccr8 via binding based on pathway neighbor associations (Kanehisa et al., 2017). Interestingly, the *Ppbp* gene was highly upregulated (fold change=14.9) in the comparison of MG1655 versus uninfected, but highly downregulated (fold change=0.2) in 278F2- versus MG1655-colonized mice. We hypothesize that regulation of the *Ppbp* gene by EHEC is a potential method used for host evasion. Future research should further characterize this interaction.

In summary, we were able to demonstrate reliable and prolonged colonization of ASF C3H/HeN mice with both a pathogenic and non-pathogenic strain of *E*. *coli*. This system can be explored in future studies to dissect the interplay between *E. coli* and members of the intestinal microbiota. Gene expression analysis showed that EHEC strain 278F2 had a modest effect on the inflammatory response in the colon of ASF mice by differentially modulating 17 genes compared to MG1655-colonized mice. Overall, these results indicate that the low-complexity ASF microbiota allowed for prolonged intestinal colonization by pathogenic and non-pathogenic strains of *E*. *coli*, and host inflammatory processes were modulated.

## MATERIALS AND METHODS

### Ethics statement

Animal experiments were carried out in accordance with the recommendations by the Guide for the Care and Use of Laboratory Animals. All experiments were performed under the approval and requirements of the Iowa State University Institutional Animal Care and Use Committee (protocols 9-04-5755-M and 1-16-8157-M). Mice were acclimated for at least 2 days before each experiment. Humane endpoint criteria were set such that any moribund animals, animals unable to feed or drink, and any animal that had greater than 10% body weight loss were euthanized immediately by carbon dioxide inhalation.

### Bacterial strains and inoculation

The non-pathogenic *E*. *coli* K-12 strain MG1655 (Blattner et al., 1997) and O157:H7 EHEC strain 278F2 isolated from a human patient (Erdem et al., 2007) obtained from Dr Jorge Girón (University of Virginia) were used throughout this work. The EHEC strain 278F2 was typed for *stx* and *eae* genes by PCR (Bai et al., 2012; Blanco et al., 2004), and further subtyped by amplicon sequencing and VirulenceFinder version 1.5 analysis and subtype-specific primers (Joensen et al., 2014; Scheutz et al., 2012). EDL933 was used as a positive control and water was used as a negative control in PCR assays. Bacterial strains were streaked on trypticase soy agar from stock cultures (−80°C) and incubated overnight at 37°C. A loopful of culture was inoculated in 3 ml of PBS until OD_600_ reached 1.0. Bacterial suspensions were diluted twofold in PBS and 0.2 ml (1×10^8^ CFU) was used for a single inoculation of mice intragastrically via oral gavage (*n*=10-13 mice per group). Dilutions of the bacterial cultures were plated on MacConkey agar to confirm the actual inoculum.

### Mice

Male and female C3H/HeN mice (12- to 15-weeks old) with a defined microbiota consisting of the ASF (originally from Taconic Biosciences, Germantown, NY) were maintained as described previously (Henderson et al., 2015). Briefly, C3H/HeN ASF mice were bred and maintained in flexible film isolators under gnotobiotic conditions following established pathogen-free husbandry practices. The ASF community has been consistently transmitted from dam to offspring and has stably colonized C3H/HeN mice for over 15 years at Iowa State University. Periodically, fecal samples have undergone 16S rRNA gene sequencing as an additional measure to ensure stability of the ASF members (Wymore Brand et al., 2015). C3H/HeN mice (12- to 15-weeks old) with a CONV microbiota were purchased from Taconic Biosciences. All inoculation and handling procedures were done with sterile instruments in a biosafety cabinet. Mice were provided an irradiated diet (Teklad 2919), autoclaved water, and a 12-h light-dark cycle was used. Animals were monitored daily for diarrhea and changes in behavior, and body weights were measured weekly. At the end of the study, mice were euthanized by carbon dioxide inhalation.

### Quantification of *E*. *coli* in feces and intestinal contents from ASF and CONV mice

Fecal samples were collected weekly over 4 weeks to determine the level of *E*. *coli* shed. Before inoculation, fecal samples were collected from ASF and CONV mice to confirm *E. coli* negative status. After 4 weeks, mice were euthanized, and contents from the duodenum, jejunum, ileum, cecum and colon were collected. To quantify *E*. *coli*, samples were weighed, homogenized in PBS, serially tenfold diluted, and plated on MacConkey agar. Concentration was determined based on CFU g^−1^ of feces or intestinal content.

### Histopathology on intestinal sections from ASF mice

Samples of the distal ileum and proximal colon (*n*=5 per group) from randomly selected ASF mice were fixed in 10% neutral buffered formalin, dehydrated through a graded alcohol and xylene series, embedded in paraffin, sectioned 5 μm thick, and stained with Hematoxylin and Eosin. Stained tissue sections were examined by a board-certified veterinary pathologist blinded to the identity of the sample. Tissues were evaluated for mucosal height and scored for epithelial injury and inflammation using a rising 5-step scale with the following criteria: 0, parameter is absent; 1, parameter is present at a low level; 2, parameter is mildly increased; 3, parameter is moderately increased; 4, parameter is severe; 5, parameter is so severe that normal architecture of the tissue is distorted or lost. Binary classification was used to describe the presence or absence of lesions observed in a given intestinal segment. Mucosal height was measured as a ratio of the length to width of the glands and only in regions of the tissue where orientation allowed for full longitudinal sections of the colonic glands to be evaluated.

### Mucosal inflammation in ASF mice

To assess mucosal inflammation, 4 days before necropsy the diet of randomly selected ASF mice (*n*=3 per group) was changed to an irradiated soy-protein-free extruded diet recommended for fluorescent optical imaging (Teklad 2920X) due to the lack of fluorescent plant material (e.g. chlorophyll). On day 27, ASF mice were injected with ProSense 680 synthesized as previously described (Bygd et al., 2015) and imaged approximately 18 h later. ProSense 680 is a near-infrared fluorescent agent that is optically silent but, in the presence of cathepsins (primarily cathepsins B, K, L and S), is cleaved at lysine-lysine sites, which in turn releases fluorophores from quenching (PerkinElmer, Waltham, MA). ProSense 680 has been validated to detect acute and chronic colonic inflammation in mice (Ding et al., 2014). The entire GI tract (stomach to rectum) was excised and images were generated using a live-animal imager (In Vivo Multispectral Imaging System FX Pro, Bruker, Billerica, MA) with a 1-min exposure and excitation and emission wavelengths of 650 and 700 nm, respectively. Images were analyzed in ImageJ with white set at a value of 0 and black a value of 65,535. An outline was drawn around each section of the intestine (stomach, small intestine, cecum or large intestine) and TCCF was measured as described previously (McCloy et al., 2014).

### Evaluation of cytokine levels in colon samples from ASF and CONV mice

Collection of colon tissues and processing was performed as previously described (Henderson et al., 2015). Briefly, a 1 cm^2^ colon tissue biopsy was collected from ASF and CONV mice (*n*=4 per group), placed in PBS to remove intestinal contents and transferred to a tube containing RPMI plus gentamicin (50 μg ml^−1^), penicillin (200 IU ml^−1^) and streptomycin (200 μg ml^−1^) at room temperature with shaking at 280 rpm. After 30 min, tissues were transferred to 96-well plates containing RPMI plus gentamicin (50 μg ml^−1^), penicillin (200 IU ml^−1^), streptomycin (200 μg ml^−1^), L-glutamine (2 mM), 2-mercaptoethanol (0.05 mM) and pyruvate (1 mM), and incubated at 37°C in 5% CO_2_. After 24 h, supernatants were harvested and frozen at −20°C until further use. Levels of proinflammatory markers IL-1β, IL-12, IFNγ and TNFα were assessed using a multiplex bead assay and Bio-Plex 200 multiplex reader (Bio-Rad, Hercules, CA).

### Eukaryotic total RNA isolation from ASF mouse colonic tissue

Samples randomly selected from uninfected, MG1655-inoculated and 278F2-inoculated ASF mice (*n*=3 per group) were used for RNA isolation. Total RNA was isolated from a dissected section of the proximal colon approximately 3 cm in length that was perforated and immersed in RNAlater (Ambion) overnight at 4°C. The next day, tissues were removed from RNAlater and stored at −80°C until further use. Tissues were homogenized for 1 min on high in a stomacher and RNA was extracted using the RNeasy Mini Kit (Qiagen). RNA integrity and concentration were assessed using the Agilent 2100 Bioanalyzer, and samples with an RNA integrity number greater than 7 were used for RNA-seq.

### Mouse inflammation panel

The isolated RNAs were sequenced on the Mouse Inflammation and Immunity Transcriptome QIAseq Targeted RNA panel (Qiagen) assaying 500 genes associated with inflammation. Count data were generated using the QIAseq Targeted RNA Panel Analysis software. Data were normalized and DEGs were determined for the contrasts between (i) uninfected versus non-pathogenic *E. coli*, (ii) uninfected versus EHEC, and (iii) non-pathogenic *E. coli* versus EHEC, using EdgeR software with default parameters with a significance threshold *P*-value<0.05 and fold-change cutoffs <0.5 and >1.5 (Table S3). Protein-protein interaction networks were created in the program STRING (Szklarczyk et al., 2015) with default parameters using DEG lists within each contrast as input data.

### Statistical analysis

All data were analyzed using GraphPad Prism version 6 software. An ANOVA followed by Tukey's test for multiple comparisons was used to compare colonization levels in fecal and intestinal samples, intestinal inflammation in ASF mice, mean body weight changes, and levels of cytokines released from colonic biopsies. The Kruskal–Wallis test was used to compare histopathology scores and Fisher's exact test (two-tailed) was used to compare proportion of tissues positive for lesions. *P*-values<0.05 were considered significant.

## Supplementary Material

Supplementary information

## References

[DMM035063C1] Adlerberth I., Lindberg E., Åberg N., Hesselmar B., Saalman R., Strannegård I.-L. and Wold A. E. (2006). Reduced enterobacterial and increased staphylococcal colonization of the infantile bowel: an effect of hygienic lifestyle? *Pediatr. Res.* 59, 96-101. 10.1203/01.pdr.0000191137.12774.b2 16380405

[DMM035063C2] Allen K. P., Randolph M. M. and Fleckenstein J. M. (2006). Importance of heat-labile enterotoxin in colonization of the adult mouse small intestine by human enterotoxigenic *Escherichia coli* strains. *Infect. Immun.* 74, 869-875. 10.1128/IAI.74.2.869-875.2006 16428729 PMC1360293

[DMM035063C3] Bäckhed F., Ding H., Wang T., Hooper L. V., Koh G. Y., Nagy A., Semenkovich C. F. and Gordon J. I. (2004). The gut microbiota as an environmental factor that regulates fat storage. *Proc. Natl. Acad. Sci. USA* 101, 15718-15723. 10.1073/pnas.0407076101 15505215 PMC524219

[DMM035063C4] Bai J., Paddock Z. D., Shi X., Li S., An B. and Nagaraja T. G. (2012). Applicability of a multiplex PCR to detect the seven major Shiga toxin-producing *Escherichia coli* based on genes that code for serogroup-specific O-antigens and major virulence factors in cattle feces. *Foodborne Pathog. Dis.* 9, 541-548. 10.1089/fpd.2011.1082 22568751

[DMM035063C5] Békássy Z. D., Calderon Toledo C., Leoj G., Kristoffersson A. C., Leopold S. R., Perez M.-T. and Karpman D. (2011). Intestinal damage in enterohemorrhagic *Escherichia coli* infection. *Pediatr. Nephrol.* 26, 2059-2071. 10.1007/s00467-010-1616-9 20809220

[DMM035063C6] Berin M. C., Darfeuille-Michaud A., Egan L. J., Miyamoto Y. and Kagnoff M. F. (2002). Role of EHEC O157:H7 virulence factors in the activation of intestinal epithelial cell NF-kappaB and MAP kinase pathways and the upregulated expression of interleukin 8. *Cell Microbiol.* 4, 635-648. 10.1046/j.1462-5822.2002.00218.x 12366401

[DMM035063C7] Blanco M., Blanco J. E., Mora A., Dahbi G., Alonso M. P., González E. A., Bernárdez M. I. and Blanco J. (2004). Serotypes, virulence genes, and intimin types of Shiga toxin (verotoxin)-producing *Escherichia coli* isolates from cattle in Spain and identification of a new intimin variant gene (*eae*-ξ). *J. Clin. Microbiol.* 42, 645-651. 10.1128/JCM.42.2.645-651.2004 14766831 PMC344521

[DMM035063C8] Blattner F. R., Plunkett G., Bloch C. A., Perna N. T., Burland V., Riley M., Collado-Vides J., Glasner J. D., Rode C. K., Mayhew G. F. et al. (1997). The complete genome sequence of *Escherichia coli* K-12. *Science* 277, 1453-1462. 10.1126/science.277.5331.1453 9278503

[DMM035063C9] Blount Z. D. (2015). The unexhausted potential of *E*. *coli*. *eLife* 4, e05826 10.7554/eLife.05826 PMC4373459 25807083

[DMM035063C10] Brando R. J. F., Miliwebsky E., Bentancor L., Deza N., Baschkier A., Ramos M. V., Fernández G. C., Meiss R., Rivas M. and Palermo M. S. (2008). Renal damage and death in weaned mice after oral infection with Shiga toxin 2-producing *Escherichia coli* strains. *Clin. Exp. Immunol.* 153, 297-306. 10.1111/j.1365-2249.2008.03698.x 18549440 PMC2492904

[DMM035063C11] Bygd H. C., Forsmark K. D. and Bratlie K. M. (2015). Altering *in vivo* macrophage responses with modified polymer properties. *Biomaterials* 56, 187-197. 10.1016/j.biomaterials.2015.03.042 25934291

[DMM035063C12] Ceponis P. J. M., McKay D. M., Ching J. C. Y., Pereira P. and Sherman P. M. (2003). Enterohemorrhagic *Escherichia coli* O157:H7 disrupts Stat1-mediated gamma interferon signal transduction in epithelial cells. *Infect. Immun.* 71, 1396-1404. 10.1128/IAI.71.3.1396-1404.2003 12595457 PMC148815

[DMM035063C13] Chen C., Blumentritt C. A., Curtis M. M., Sperandio V., Torres A. G. and Dudley E. G. (2013). Restrictive streptomycin resistance mutations decrease the formation of attaching and effacing lesions in *Escherichia coli* O157:H7 strains. *Antimicrob. Agents Chemother.* 57, 4260-4266. 10.1128/AAC.00709-13 23796920 PMC3754346

[DMM035063C14] Cheng Y.-L., Song L.-Q., Huang Y.-M., Xiong Y.-W., Zhang X.-A., Sun H., Zhu X.-P., Meng G.-X., Xu J.-G. and Ren Z.-H. (2015). Effect of enterohaemorrhagic *Escherichia coli* O157:H7-specific enterohaemolysin on interleukin-1β production differs between human and mouse macrophages due to the different sensitivity of NLRP3 activation. *Immunology* 145, 258-267. 10.1111/imm.12442 25580516 PMC4427390

[DMM035063C15] Cole D., Griffin P. M., Fullerton K. E., Ayers T., Smith K., Ingram L. A., Kissler B. and Hoekstra R. M. (2014). Attributing sporadic and outbreak-associated infections to sources: blending epidemiological data. *Epidemiol. Infect.* 142, 295-302. 10.1017/S0950268813000915 23611460 PMC9151115

[DMM035063C16] Conlan J. W. and Perry M. B. (1998). Susceptibility of three strains of conventional adult mice to intestinal colonization by an isolate of *Escherichia coli* O157:H7. *Can. J. Microbiol.* 44, 800-805. 10.1139/w98-056 9830109

[DMM035063C17] Conway T. and Cohen P. S. (2015). Commensal and pathogenic *Escherichia coli* metabolism in the gut. *Microbiol. Spectr.* 3 10.1128/microbiolspec.MBP-0006-2014 PMC4510460 26185077

[DMM035063C18] Croxen M. A., Law R. J., Scholz R., Keeney K. M., Wlodarska M. and Finlay B. B. (2013). Recent advances in understanding enteric pathogenic *Escherichia coli*. *Clin. Microbiol. Rev.* 26, 822-880. 10.1128/CMR.00022-13 24092857 PMC3811233

[DMM035063C19] Ding S., Blue R. E., Morgan D. R. and Lund P. K. (2014). Comparison of multiple enzyme activatable near-infrared fluorescent molecular probes for detection and quantification of inflammation in murine colitis models. *Inflamm. Bowel Dis.* 20, 363-377. 10.1097/01.MIB.0000440612.98950.79 24374874 PMC4618379

[DMM035063C20] Eaton K. A., Friedman D. I., Francis G. J., Tyler J. S., Young V. B., Haeger J., Abu-Ali G. and Whittam T. S. (2008). Pathogenesis of renal disease due to enterohemorrhagic *Escherichia coli* in germ-free mice. *Infect. Immun.* 76, 3054-3063. 10.1128/IAI.01626-07 18443087 PMC2446693

[DMM035063C21] Eaton K. A., Fontaine C., Friedman D. I., Conti N. and Alteri C. J. (2017). Pathogenesis of colitis in germ-free mice infected with EHEC O157:H7. *Vet. Pathol.* 54, 710-719. 10.1177/0300985817691582 28178427 PMC5474182

[DMM035063C22] Eggesbø M., Moen B., Peddada S., Baird D., Rugtveit J., Midtvedt T., Bushel P. R., Sekelja M. and Rudi K. (2011). Development of gut microbiota in infants not exposed to medical interventions. *APMIS* 119, 17-35. 10.1111/j.1600-0463.2010.02688.x 21143523 PMC3058492

[DMM035063C23] Erdem A. L., Avelino F., Xicohtencatl-Cortes J. and Girón J. A. (2007). Host protein binding and adhesive properties of H6 and H7 flagella of attaching and effacing *Escherichia coli*. *J. Bacteriol.* 189, 7426-7435. 10.1128/JB.00464-07 17693516 PMC2168434

[DMM035063C24] Feng P., Lampel K. A., Karch H. and Whittam T. S. (1998). Genotypic and phenotypic changes in the emergence of *Escherichia coli* O157:H7. *J. Infect. Dis.* 177, 1750-1753. 10.1086/517438 9607864

[DMM035063C25] Francis D. H., Collins J. E. and Duimstra J. R. (1986). Infection of gnotobiotic pigs with an *Escherichia coli* O157:H7 strain associated with an outbreak of hemorrhagic colitis. *Infect. Immun.* 51, 953-956. 3512443 10.1128/iai.51.3.953-956.1986 PMC260992

[DMM035063C26] Francis D. H., Moxley R. A. and Andraos C. Y. (1989). Edema disease-like brain lesions in gnotobiotic piglets infected with *Escherichia coli* serotype O157:H7. *Infect. Immun.* 57, 1339-1342. 2647636 10.1128/iai.57.4.1339-1342.1989 PMC313274

[DMM035063C27] Gao X., Wan F., Mateo K., Callegari E., Wang D., Deng W., Puente J., Li F., Chaussee M. S., Finlay B. B. et al. (2009). Bacterial effector binding to ribosomal protein s3 subverts NF-kappaB function. *PLoS Pathog.* 5, e1000708 10.1371/journal.ppat.1000708 20041225 PMC2791202

[DMM035063C28] Gauger E. J., Leatham M. P., Mercado-Lubo R., Laux D. C., Conway T. and Cohen P. S. (2007). Role of motility and the *flhDC* operon in *Escherichia coli* MG1655 colonization of the mouse intestine. *Infect. Immun.* 75, 3315-3324. 10.1128/IAI.00052-07 17438023 PMC1932950

[DMM035063C29] Geuking M. B., Cahenzli J., Lawson M. A. E., Ng D. C. K., Slack E., Hapfelmeier S., McCoy K. D. and Macpherson A. J. (2011). Intestinal bacterial colonization induces mutualistic regulatory T cell responses. *Immunity* 34, 794-806. 10.1016/j.immuni.2011.03.021 21596591

[DMM035063C30] Goswami K., Chen C., Xiaoli L., Eaton K. A. and Dudley E. G. (2015). Coculture of *Escherichia coli* O157:H7 with a nonpathogenic *E*. *coli* strain increases toxin production and virulence in a germfree mouse model. *Infect. Immun.* 83, 4185-4193. 10.1128/IAI.00663-15 26259815 PMC4598395

[DMM035063C31] Gould L. H., Mody R. K., Ong K. L., Clogher P., Cronquist A. B., Garman K. N., Lathrop S., Medus C., Spina N. L., Webb T. H. et al. (2013). Increased recognition of non-O157 Shiga toxin-producing *Escherichia coli* infections in the United States during 2000-2010: epidemiologic features and comparison with *E*. *coli* O157 infections. *Foodborne Pathog. Dis.* 10, 453-460. 10.1089/fpd.2012.1401 23560425

[DMM035063C32] Gradel K. O., Nielsen H. L., Schønheyder H. C., Ejlertsen T., Kristensen B. and Nielsen H. (2009). Increased short- and long-term risk of inflammatory bowel disease after *Salmonella* or *Campylobacter* gastroenteritis. *Gastroenterology* 137, 495-501. 10.1053/j.gastro.2009.04.001 19361507

[DMM035063C33] Gyles C. L. (2007). Shiga toxin-producing *Escherichia coli*: an overview. *J. Anim. Sci.* 85, E45-E62. 10.2527/jas.2006-508 17085726

[DMM035063C34] Hauf N. and Chakraborty T. (2003). Suppression of NF-kappa B activation and proinflammatory cytokine expression by Shiga toxin-producing *Escherichia coli*. *J. Immunol.* 170, 2074-2082. 10.4049/jimmunol.170.4.2074 12574378

[DMM035063C35] Hayashi T., Makino K., Ohnishi M., Kurokawa K., Ishii K., Yokoyama K., Han C. G., Ohtsubo E., Nakayama K., Murata T. et al. (2001). Complete genome sequence of enterohemorrhagic *Escherichia coli* O157:H7 and genomic comparison with a laboratory strain K-12. *DNA Res.* 8, 11-22. 10.1093/dnares/8.1.11 11258796

[DMM035063C36] Henderson A. L., Brand M. W., Darling R. J., Maas K. J., Detzel C. J., Hostetter J., Wannemuehler M. J. and Weaver E. M. (2015). Attenuation of colitis by serum-derived bovine immunoglobulin/protein isolate in a defined microbiota mouse model. *Dig. Dis. Sci.* 60, 3293-3303. 10.1007/s10620-015-3726-5 26026602 PMC4621698

[DMM035063C37] Hong S., Oh K.-H., Cho S.-H., Kim J.-C., Park M.-S., Lim H.-S. and Lee B.-K. (2009). Asymptomatic healthy slaughterhouse workers in South Korea carrying Shiga toxin-producing *Escherichia coli*. *FEMS Immunol. Med. Microbiol.* 56, 41-47. 10.1111/j.1574-695X.2009.00545.x 19309486

[DMM035063C38] Hooper L. V., Littman D. R. and Macpherson A. J. (2012). Interactions between the microbiota and the immune system. *Science* 336, 1268-1273. 10.1126/science.1223490 22674334 PMC4420145

[DMM035063C39] Jandu N., Ceponis P. J. M., Kato S., Riff J. D., McKay D. M. and Sherman P. M. (2006). Conditioned medium from enterohemorrhagic *Escherichia coli*-infected T84 cells inhibits signal transducer and activator of transcription 1 activation by gamma interferon. *Infect. Immun.* 74, 1809-1818. 10.1128/IAI.74.3.1809-1818.2006 16495555 PMC1418659

[DMM035063C40] Jandu N., Shen S., Wickham M. E., Prajapati R., Finlay B. B., Karmali M. A. and Sherman P. M. (2007). Multiple seropathotypes of verotoxin-producing *Escherichia coli* (VTEC) disrupt interferon-gamma-induced tyrosine phosphorylation of signal transducer and activator of transcription (Stat)-1. *Microb. Pathog.* 42, 62-71. 10.1016/j.micpath.2006.10.005 17174521

[DMM035063C41] Joensen K. G., Scheutz F., Lund O., Hasman H., Kaas R. S., Nielsen E. M. and Aarestrup F. M. (2014). Real-time whole-genome sequencing for routine typing, surveillance, and outbreak detection of verotoxigenic *Escherichia coli*. *J. Clin. Microbiol.* 52, 1501-1510. 10.1128/JCM.03617-13 24574290 PMC3993690

[DMM035063C42] Kanehisa M., Furumichi M., Tanabe M., Sato Y. and Morishima K. (2017). KEGG: new perspectives on genomes, pathways, diseases and drugs. *Nucleic Acids Res.* 45, D353-D361. 10.1093/nar/gkw1092 27899662 PMC5210567

[DMM035063C43] Kang G., Pulimood A. B., Koshi R., Hull A., Acheson D., Rajan P., Keusch G. T., Mathan V. I. and Mathan M. M. (2001). A monkey model for enterohemorrhagic *Escherichia coli* infection. *J. Infect. Dis.* 184, 206-210. 10.1086/322011 11424020

[DMM035063C44] Karpman D., Connell H., Svensson M., Scheutz F., Aim P. and Svanborg C. (1997). The role of lipopolysaccharide and Shiga-like toxin in a mouse model of *Escherichia coli* O157:H7 infection. *J. Infect. Dis.* 175, 611-620. 10.1093/infdis/175.3.611 9041333

[DMM035063C45] Karve S. S., Pradhan S., Ward D. V. and Weiss A. A. (2017). Intestinal organoids model human responses to infection by commensal and Shiga toxin producing *Escherichia coli*. *PLoS ONE* 12, e0178966 10.1371/journal.pone.0178966 28614372 PMC5470682

[DMM035063C46] Kim Y., Oh S., Park S. and Kim S. H. (2009). Interactive transcriptome analysis of enterohemorrhagic *Escherichia coli* (EHEC) O157:H7 and intestinal epithelial HT-29 cells after bacterial attachment. *Int. J. Food Microbiol.* 131, 224-232. 10.1016/j.ijfoodmicro.2009.03.002 19336271

[DMM035063C47] Leatham M. P., Banerjee S., Autieri S. M., Mercado-Lubo R., Conway T. and Cohen P. S. (2009). Precolonized human commensal *Escherichia coli* strains serve as a barrier to *E*. *coli* O157:H7 growth in the streptomycin-treated mouse intestine. *Infect. Immun.* 77, 2876-2886. 10.1128/IAI.00059-09 19364832 PMC2708557

[DMM035063C48] Lindgren S. W., Melton A. R. and O'Brien A. D. (1993). Virulence of enterohemorrhagic *Escherichia coli* O91:H21 clinical isolates in an orally infected mouse model. *Infect. Immun.* 61, 3832-3842. 8359904 10.1128/iai.61.9.3832-3842.1993 PMC281084

[DMM035063C49] Mahalingam S. and Karupiah G. (1999). Chemokines and chemokine receptors in infectious diseases. *Immunol. Cell Biol.* 77, 469-475. 10.1046/j.1440-1711.1999.00858.x 10571666

[DMM035063C50] McCloy R. A., Rogers S., Caldon C. E., Lorca T., Castro A. and Burgess A. (2014). Partial inhibition of Cdk1 in G 2 phase overrides the SAC and decouples mitotic events. *Cell Cycle* 13, 1400-1412. 10.4161/cc.28401 24626186 PMC4050138

[DMM035063C51] Miliwebsky E., Deza N., Chinen I., Martinez Espinosa E., Gomez D., Pedroni E., Caprile L., Bashckier A., Manfredi E., Leotta G. et al. (2007). Prolonged fecal shedding of Shiga toxin-producing *Escherichia coli* among children attending day-care centers in Argentina. *Rev. Argent. Microbiol.* 39, 90-92. 17702253

[DMM035063C52] Mohawk K. L. and O'Brien A. D. (2011). Mouse models of *Escherichia coli* O157:H7 infection and Shiga toxin injection. *J. Biomed. Biotechnol.* 2011, 258185 10.1155/2011/258185 21274267 PMC3022220

[DMM035063C53] Mohawk K. L., Melton-Celsa A. R., Zangari T., Carroll E. E. and O'Brien A. D. (2010). Pathogenesis of *Escherichia coli* O157:H7 strain 86-24 following oral infection of BALB/c mice with an intact commensal flora. *Microb. Pathog.* 48, 131-142. 10.1016/j.micpath.2010.01.003 20096770 PMC2834854

[DMM035063C54] Nadler C., Baruch K., Kobi S., Mills E., Haviv G., Farago M., Alkalay I., Bartfeld S., Meyer T. F., Ben-Neriah Y. et al. (2010). The type III secretion effector NleE inhibits NF-kappaB activation. *PLoS Pathog.* 6, e1000743 10.1371/journal.ppat.1000743 20126447 PMC2813277

[DMM035063C55] Nagano K., Taguchi K., Hara T., Yokoyama S., Kawada K. and Mori H. (2003). Adhesion and colonization of enterohemorrhagic *Escherichia coli* O157:H7 in cecum of mice. *Microbiol. Immunol.* 47, 125-132. 10.1111/j.1348-0421.2003.tb02795.x 12680715

[DMM035063C56] O'Donnell J. M., Thornton L., McNamara E. B., Prendergast T., Igoe D. and Cosgrove C. (2002). Outbreak of Vero cytotoxin-producing *Escherichia coli* O157 in a child day care facility. *Commun. Dis. Public Health* 5, 54-58. 12070979

[DMM035063C57] Pearson J. S. and Hartland E. L. (2014). The inflammatory response during enterohemorrhagic *Escherichia coli* infection. *Microbiol. Spectr.* 2, EHEC-0012-2013 10.1128/microbiolspec.EHEC-0012-2013 26104206

[DMM035063C58] Raife T., Friedman K. D. and Fenwick B. (2004). Lepirudin prevents lethal effects of Shiga toxin in a canine model. *Thromb. Haemost.* 92, 387-393. 10.1160/TH03-12-0759 15269836

[DMM035063C59] Roxas J. L., Koutsouris A., Bellmeyer A., Tesfay S., Royan S., Falzari K., Harris A., Cheng H., Rhee K. J. and Hecht G. (2010). Enterohemorrhagic *E*. *coli* alters murine intestinal epithelial tight junction protein expression and barrier function in a Shiga toxin independent manner. *Lab. Invest.* 90, 1152-1168. 10.1038/labinvest.2010.91 20479715 PMC2912457

[DMM035063C60] Sarma-Rupavtarm R. B., Ge Z., Schauer D. B., Fox J. G. and Polz M. F. (2004). Spatial distribution and stability of the eight microbial species of the altered Schaedler flora in the mouse gastrointestinal tract. *Appl. Environ. Microbiol.* 70, 2791-2800. 10.1128/AEM.70.5.2791-2800.2004 15128534 PMC404395

[DMM035063C61] Scheithauer T. P. M., Dallinga-Thie G. M., de Vos W. M., Nieuwdorp M. and van Raalte D. H. (2016). Causality of small and large intestinal microbiota in weight regulation and insulin resistance. *Mol. Metab.* 5, 759-770. 10.1016/j.molmet.2016.06.002 27617199 PMC5004227

[DMM035063C62] Scheutz F., Teel L. D., Beutin L., Piérard D., Buvens G., Karch H., Mellmann A., Caprioli A., Tozzoli R., Morabito S. et al. (2012). Multicenter evaluation of a sequence-based protocol for subtyping Shiga toxins and standardizing Stx nomenclature. *J. Clin. Microbiol.* 50, 2951-2963. 10.1128/JCM.00860-12 22760050 PMC3421821

[DMM035063C63] Shah S., Hoffman R., Shillam P. and Wilson B. (1996). Prolonged fecal shedding of *Escherichia coli* O157:H7 during an outbreak at a day care center. *Clin. Infect. Dis.* 23, 835-836. 10.1093/clinids/23.4.835 8909859

[DMM035063C64] Silvestro L., Caputo M., Blancato S., Decastelli L., Fioravanti A., Tozzoli R., Morabito S. and Caprioli A. (2004). Asymptomatic carriage of verocytotoxin-producing *Escherichia coli* O157 in farm workers in Northern Italy. *Epidemiol. Infect.* 132, 915-919. 10.1017/S0950268804002390 15473155 PMC2870179

[DMM035063C65] Small C. L., Xing L., McPhee J. B., Law H. T. and Coombes B. K. (2016). Acute infectious gastroenteritis potentiates a Crohn's disease pathobiont to fuel ongoing inflammation in the post-infectious period. *PLoS Pathog.* 12, e1005907 10.1371/journal.ppat.1005907 27711220 PMC5053483

[DMM035063C66] Spees A. M., Wangdi T., Lopez C. A., Kingsbury D. D., Xavier M. N., Winter S. E., Tsolis R. M. and Bäumler A. J. (2013). Streptomycin-induced inflammation enhances *Escherichia coli* gut colonization through nitrate respiration. *MBio* 4, e00430-13 10.1128/mBio.00430-13 PMC3705454 23820397

[DMM035063C67] Stecher B., Chaffron S., Käppeli R., Hapfelmeier S., Freedrich S., Weber T. C., Kirundi J., Suar M., McCoy K. D., von Mering C. et al. (2010). Like will to like: abundances of closely related species can predict susceptibility to intestinal colonization by pathogenic and commensal bacteria. *PLoS Pathog.* 6, e1000711 10.1371/journal.ppat.1000711 20062525 PMC2796170

[DMM035063C68] Stevens M. P. and Frankel G. M. (2014). The locus of enterocyte effacement and associated virulence factors of enterohemorrhagic *Escherichia coli*. *Microbiol. Spectr.* 2 10.1128/microbiolspec.EHEC-0007-2013 26104209

[DMM035063C69] Szebeni G. J., Vizler C., Kitajka K. and Puskas L. G. (2017). Inflammation and cancer: extra- and intracellular determinants of tumor-associated macrophages as tumor promoters. *Mediators Inflamm.* 2017, 9294018 10.1155/2017/9294018 28197019 PMC5286482

[DMM035063C70] Szklarczyk D., Franceschini A., Wyder S., Forslund K., Heller D., Huerta-Cepas J., Simonovic M., Roth A., Santos A., Tsafou K. P. et al. (2015). STRING v10: protein-protein interaction networks, integrated over the tree of life. *Nucleic Acids Res.* 43, D447-D452. 10.1093/nar/gku1003 25352553 PMC4383874

[DMM035063C71] Taguchi H., Takahashi M., Yamaguchi H., Osaki T., Komatsu A., Fujioka Y. and Kamiya S. (2002). Experimental infection of germ-free mice with hyper-toxigenic enterohaemorrhagic *Escherichia coli* O157:H7, strain 6. *J. Med. Microbiol.* 51, 336-343. 10.1099/0022-1317-51-4-336 11926740

[DMM035063C72] Tarr P. I., Gordon C. A. and Chandler W. L. (2005). Shiga-toxin-producing *Escherichia coli* and haemolytic uraemic syndrome. *Lancet* 365, 1073-1086. 10.1016/S0140-6736(05)71144-2 15781103

[DMM035063C73] Taylor F. B., Tesh V. L., DeBault L., Li A., Chang A. C. K., Kosanke S. D., Pysher T. J. and Siegler R. L. (1999). Characterization of the baboon responses to Shiga-like toxin: descriptive study of a new primate model of toxic responses to Stx-1. *Am. J. Pathol.* 154, 1285-1299. 10.1016/S0002-9440(10)65380-1 10233866 PMC1866558

[DMM035063C74] Trinchieri G. (2003). Interleukin-12 and the regulation of innate resistance and adaptive immunity. *Nat. Rev. Immunol.* 3, 133-146. 10.1038/nri1001 12563297

[DMM035063C75] Tzipori S., Wachsmuth I. K., Chapman C., Birner R., Brittingham J., Jackson C. and Hogg J. (1986). The pathogenesis of hemorrhagic colitis caused by *Escherichia coli* O157:H7 in gnotobiotic piglets. *J. Infect. Dis.* 154, 712-716. 10.1093/infdis/154.4.712 3528323

[DMM035063C76] Tzipori S., Montanaro J., Robins-Browne R. M., Vial P., Gibson R. and Levine M. M. (1992). Studies with enteroaggregative *Escherichia coli* in the gnotobiotic piglet gastroenteritis model. *Infect. Immun.* 60, 5302-5306. 10.1016/S0140-6736(05)71144-2 1452364 PMC258311

[DMM035063C77] Wymore Brand M., Wannemuehler M. J., Phillips G. J., Proctor A., Overstreet A.-M., Jergens A. E., Orcutt R. P. and Fox J. G. (2015). The altered Schaedler flora: continued applications of a defined murine microbial community. *ILAR J.* 56, 169-178. 10.1093/ilar/ilv012 26323627 PMC4554250

