# Supplementary material for: Pathogenic and non-pathogenic Escherichia coli colonization and host inflammatory response in a defined microbiota mouse model
Source: Dis Model Mech. 2018 Nov 16;11(11):dmm035063. doi: 10.1242/dmm.035063 (PMC6262807; doi:10.1242/dmm.035063)
Supplement: Supplementary information [file dmm-11-035063-s1.pdf]

Supplementary Information

Table S1. Body weight changes of mice harboring the altered Schaedler flora (ASF) or a conventionally-reared (CONV) microbiota after gavage with PBS, non-pathogenic *Escherichia coli* MG1655, or enterohemorrhagic *E. coli* 278F2

**Group**	**Mean body weight in grams (Mean percent change in body weight)**			
	**Day 7**	**Day 14**	**Day 21**	**Day 27**
Uninfected ASF mice	29.2 (0.8)	29.3 (1.9)	29.8 (3.0[a])	31.2 (7.8[a])
Uninfected CONV mice	30.9 (1.1)	33.4 (7.6)	35.4 (14.6[b])	36.5 (18.6[b])
MG1655 ASF mice	31.4 (-3.4)	33.1 (1.6)	33.5 (3.0[a])	34.3 (5.5[a])
MG1655 CONV mice	29.8 (3.4)	32.2 (9.8)	33.4 (14.2[b])	34.7 (18.6[b])
278F2 ASF mice	28.8 (-0.6)	29.9 (3.4)	30.6 (5.7[ac])	31.2 (7.8[a])
278F2 CONV mice	30.0 (2.2)	30.5 (4.6)	33.1 (13.4[bc])	34.1 (16.9[b])

Mice within day post-inoculation were compared by an ANOVA followed by Tukey's method for multiple means comparison for mean body weight and mean percent change in body weight. Means with the same letter were not significantly different ($P > 0.05$).

Disease Models & Mechanisms • Supplementary information

Table S2. RNA quality and sequences generated pre- and post-filtering

Sample no.	Inoculation[a]	RIN	No. of sequences pre-filtering	No. of sequences post-filtering
1	Uninfected	10	1,647,561	1,576,794
2	Uninfected	9.9	1,523,570	1,460,963
3	Uninfected	8.1	1,572,396	1,488,989
4	MG1655	9.3	1,769,987	1,690,408
5	MG1655	7.8	1,423,855	1,361,385
6	MG1655	10	1,731,238	1,644,878
7	278F2	8.4	2,730,355	2,606,733
8	278F2	9.7	1,323,237	1,261,961
9	278F2	8.4	1,526,550	1,456,184

RIN, RNA integrity number.

[a]Uninfected indicates PBS, MG1655 indicates the non-pathogenic *Escherichia coli* strain, and 278F2 indicates the enterohemorrhagic *E. coli* strain.

Table S3. RNAseq data for the comparisons of non-pathogenic *Escherichia coli* MG1655 versus uninfected, enterohemorrhagic *E. coli* 278F2 versus uninfected, and enterohemorrhagic *E. coli* 278F2 versus non-pathogenic *E. coli* MG1655

Click here to Download Table S3

Disease Models & Mechanisms • Supplementary information

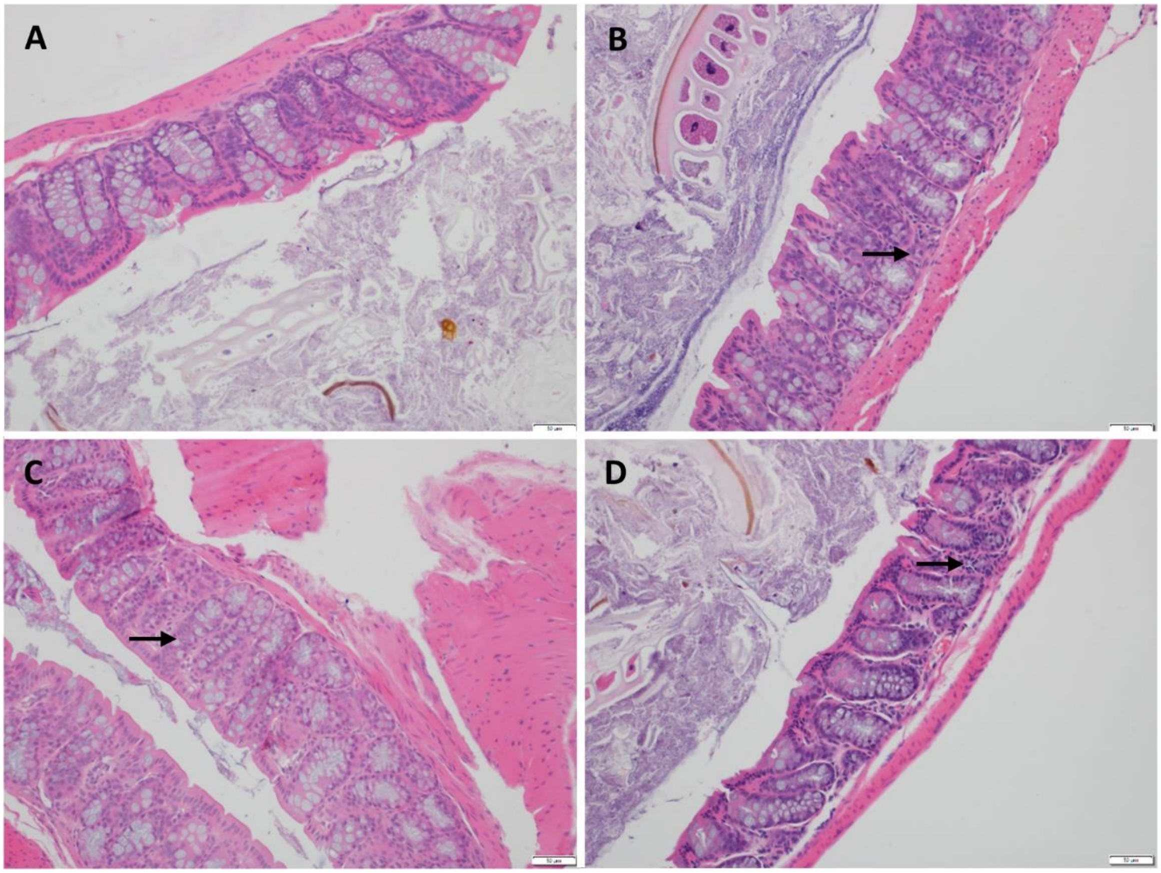

Fig. S1. Microscopic evaluation of colonic tissue from altered Schaedler flora (ASF) mice. Photomicrographs of colons from (A) uninfected ASF mice showing normal architecture and lesions (arrows) in (B) non-pathogenic *Escherichia coli* MG1655 colonized mice showing increased inflammatory cells (arrow), and enterohemorrhagic *E. coli* 278F2 colonized ASF mice with (C) elongation of the glands (arrow), and (D) increased inflammatory cells (arrow).

Disease Models & Mechanisms • Supplementary information

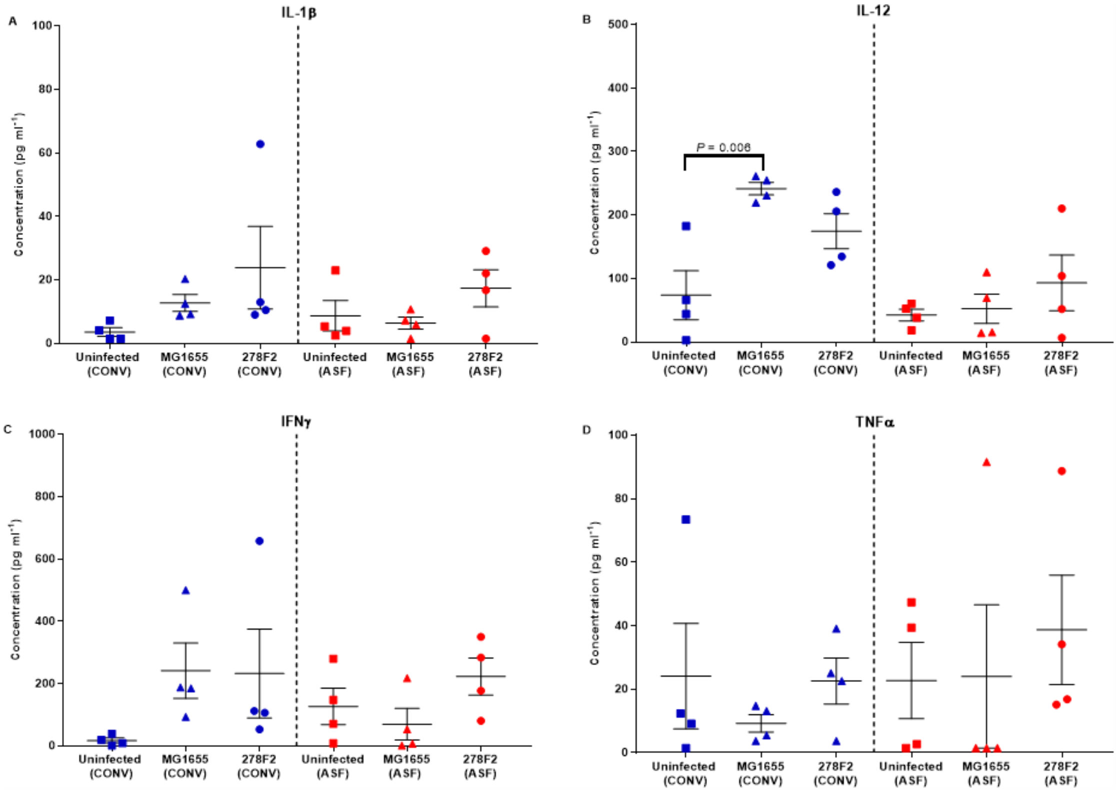

Fig. S2. Levels of proinflammatory cytokines released from colonic biopsies. Colon tissue samples (n = 4 per group) were obtained 28 days post-inoculation from uninfected, non-pathogenic *Escherichia coli* MG1655 inoculated, and enterohemorrhagic *E. coli* 278F2 inoculated altered Schaedler flora (ASF) and conventionally-reared (CONV) mice. Secretion of (A) IL-1β, (B) IL-12, (C) interferon gamma (IFNγ), and (D) tumor necrosis factor alpha (TNFα) was elevated. Results are shown as mean±s.e.m. and each symbol represents an individual animal. Blue and red symbols represent CONV and ASF mice, respectively. An ANOVA followed by Tukey's method for multiple means comparison was used to compare cytokine levels within ASF or CONV mice.
